# Impact of NLRP3 gene polymorphisms (rs10754558 and rs10733113) on HPV infection and cervical cancer in southern Chinese population

**DOI:** 10.1186/s13027-023-00529-4

**Published:** 2023-10-26

**Authors:** Qingchun Lu, Xiaoxia Lao, Jinghua Gan, Ping Du, Yingpei Zhou, Wenzheng Nong, Zhige Yang

**Affiliations:** 1 Department of Gynecology, Minzu Hospital of Guangxi Zhuang Autonomous Region, Affiliated Minzu Hospital of Guangxi Medical University, Guangxi, China; 2 Department of Clinical Laboratory, Minzu Hospital of Guangxi Zhuang Autonomous Region, Affiliated Minzu Hospital of Guangxi Medical University, Guangxi, China

**Keywords:** NLRP3, Inflammasome, Polymorphism, Cervical cancer, HPV, Cervical intraepithelial neoplasia

## Abstract

**Objective:**

Mutations in the NLRP3gene have previously been linked to certain forms of cancer, but there have not been any specific studies examining the association between NLRP3 polymorphisms and cervical cancer (CC). This study was therefore designed to investigate the effect of NLRP3 gene polymorphisms on HPV infection and cervical cancer in southern Chinese population.

**Methods:**

Multiplex PCR and next-generation sequencing approaches were used to assess the NLRP3 rs10754558 and rs10733113 polymorphisms in 404 cervical lesion patients, including 227 diagnosed with CC and 177 diagnosed with cervical intraepithelial neoplasia(CIN), with 419 healthy female controls being included for comparison. Correlations between the rs10754558 and rs10733113 genotypes and alleles in these patients and CC and CIN were then analyzed.

**Results:**

No correlations were found between NLRP3 rs10754558 and rs10733113 and human papillomavirus(HPV) infection status. Relative to the healthy control group, the NLRP3 rs10754558 GG genotype, CG + GG genotype, and G allele frequencies were significantly increased among patients with cervical lesions (CC and CIN) (OR = 1.815,P = 0.013;OR = 1.383, P = 0.026; OR = 1.284, P = 0.014,respectively), whereas no such differences were observed for rs10733113. A higher cervical lesion risk was detected for patients over the age of 45 exhibiting the rs10754558 GG genotype (OR = 1.848, P = 0.040). Additionally, the risk of CC was elevated in patients with the rs10754558 GG genotype or the G allele relative to patients with the CC genotype or the C allele(OR = 1.830, P = 0.029; OR = 1.281, P = 0.039). The rs10733113 genotypes or alleles were not significantly associated with CC risk (P > 0.05). No association between rs10754558 and rs10733113 genotypes and CC patient clinicopathological features were observed (P > 0.05). Serum NLRP3, IL-1β, and IL-18 levels were significantly elevated in CC patients relative to healthy controls(P < 0.05). Relative to the CC genotype, CC patients harboring the rs10754558 GG genotype exhibited significantly elevated IL-1β and IL-18 levels(P < 0.05).

**Conclusion:**

The rs10754558 polymorphism in the NLRP3 gene may contribute to an elevated risk of CC, although it is not significantly correlated with HPV infection and CC progression.

## Introduction

Cervical cancer (CC) is among the most prevalent forms of gynecological cancer, with an estimated 600,000 diagnoses and 340,000 deaths associated with this cancer type throughout the world annually [1]. CC incidence occurs as a result of a range of factors and genetic interactions, proceeding through a series of steps from initial precancerous cervical intraepithelial neoplasia (CIN) lesions to malignant tumor development. The pathogenesis of CC is highly complex, and high-risk human papillomavirus (HPV) infection is the risk factor that is most closely associated with higher odds of developing this form of cancer. However, just 10% of women affected by HPV ultimately develop precancerous lesions, and under 1% of these lesions progress to CC [2]. Many factors closely associated with individual CC susceptibility have been identified to date, including sexual activity, immune status, and the presence of certain genetic polymorphisms [3]. As the most common type of heritable genetic variation, single nucleotide polymorphisms (SNPs) can impact the transcription and expression of particular genes in a manner that may ultimately shape the risk of oncogenesis [4]. Specific genetic variants can impact HPV infection susceptibility or persistence, thereby indirectly influencing tumor development rates [5]. Studies of the association between specific SNPs and CC risk have thus been a focus of exhaustive research interest, highlighting a range of promising opportunities for preventative or therapeutic intervention in some cases.

Inflammatory activity serves as an essential mediator of key oncogenic processes including malignant transformation, angiogenesis, disease progression, and the invasion of local and distant tissues [6, 7]. The Nod-like receptor protein 3(NLRP3) inflammasome is the best-characterized inflammasome complex, and it can be activated in response to both damage- and pathogen- associated molecular patterns in specific contexts. NLRP3 inflammasome activity is associated with key tumorigenesis-related processes. Functionally, the activation of the NLRP3 inflammasome can result in the pyroptotic programmed death of cells and the release of the proinflammatory cytokines IL-1β and IL-18, which can establish inflammatory microenvironmental conditions conducive to tumor growth [8]. Owing to these two potentially opposing activities, NLRP3 inflammasome activity has been characterized as a “double-edged sword” within tumors [9], with a range of studies having ascribed pro- and anti-tumorignenic roles to NLRP3 in particular tumor types.

NLRP3 is encoded by a 30 kb gene on chromosome 1q44 consisting of 8 introns and 9 exons. To date, roughly 60 SNPs in the NLRP3 gene have been reported. These variants can alter the function and structure of the encoded NLRP3 protein, with some resulting in its persistent activation, in turn driving extensive pro-inflammatory cytokine secretion and the induction of a robust inflammatory response. NLRP3 polymorphisms have been linked to increased susceptibility to disease including certain forms of cardiovascular disease, autoimmunity, and malignancies [10–12]. The rs10754558 polymorphism in the 3’-untranslated region (UTR) of the NLRP3 gene can impact the stability and translation of the resultant mRNA [13], and it has thus been the subject of extensive research interest. This SNP has been linked to many different diseases including diabetes, COVID-19, and bladder cancer [14–16]. The rs10733113 polymorphism in the NLRP3 gene has also been studied in the context of inflammatory bowel disease [17] and psoriatic arthritis [18], although its relationship with cancer remains to be studied at length. The association between the NLRP3 rs10754558 and rs10733113 polymorphisms and CC risk remains to be characterized. Accordingly, in this study, the impact of the rs1075455 and rs10733113 polymorphisms in the NLRP3 gene on HPV infection and CC risk was explored in southern Chinese population.

## Materials and methods

### Study subjects

All patients were recruited from the Department of Gynecology of Minzu Hospital of Guangxi Zhuang Autonomous Region, Affiliated with Minzu Hospital of Guangxi Medical University (Guangxi, China) between January 2019 and June 2022. Cases included 404 cervical lesions, including 227 CC patients and 177 CIN patients. Pathological experts from Minzu Hospital of Guangxi Zhuang Autonomous Region confirmed all patient diagnoses. Patients were excluded if: (1) they had undergone treatment prior to sample collection, (2) they exhibited benign cervical lesions, benign cervical tumors, or other types of malignant tumors, (3) they exhibited chronic diseases including diabetes or cardiovascular disease, or (4) they had been diagnosed with other acute or chronic infections or related diseases. As a control group, 419 healthy females were recruited during this same period through thephysical examination center of the same hospital. The Ethics Committee of Minzu Hospital of Guangxi Zhuang Autonomous Region approved this study.

### DNA isolation and genotyping

**Fig. 1:**
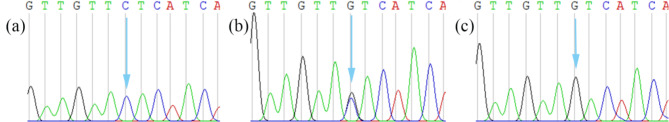
Sequencing map of the genotype for the NLRP3 rs10754558 polymorphism. Arrow in parts (**a**-**c**) indicates CC, C/G and GG genotypes, respectively

**Fig. 2:**
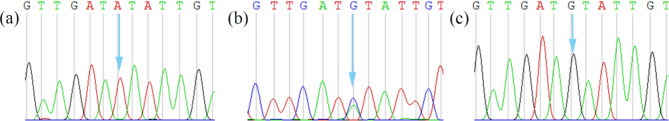
Sequencing map of the genotype for the NLRP3 rs10733113 polymorphism. Arrow in parts (**a**-**c**) indicates AA, G/A and GG genotypes, respectively

A 2 mL sample of venous blood was collected from each study participant and stored at -80℃prior to use. A TIANamp Genomic DNA Kit (Tiangen Biotech, Beijing, China) was used based on provided directions to extract DNA from these peripheral blood samples, after which multiplex PCR and next-generation sequencing of the rs10754558 and rs10733113 polymorphisms in the NLRP3 gene was performed for each sample. Briefly, DNA concentration and purity were assessed, and agarose gel electrophoresis was performed to confirm that DNA bands were free of any impurities or other indicators of poor DNA quality. PCR was then performed with primers synthesized by Qike Biotechnology (Beijing,China) and the reaction conditions were showed in Table 1. After electrophoretic confirmation of sequencing products, they were sent to Qike Biotechnology (Guangzhou, China) for sequencing and genotyping (Figs. 1 and 2).

**Table 1:** Primer Sequence and the Reaction Condition for NLRP3 Polymorphisms

SNP	Primer Sequence	PCR Reaction Conditions			Length
		Initial Step	Melt/Anneal/Extend	Elongate Step	
rs10754558	Forward:5’-ACCCAGGCTTTCTATTTGCTTT-3’	94℃ for 3 min	35 cycles of 94℃ for 30 s, 57℃ for 30s, and 72℃ for 30 s	72℃ for 5 min	402 bp
	Reverse:5’-ATGAGGTCACCAAGAGGAACATT-3’				
rs10733113	Forward:5’-AACCTATCTGTGCCTCACTTGT − 3’				248 bp
	Reverse: 5’-ACTACTTCTTGCGGCCTGTC − 3’				

### Serum NLRP3, IL-1β, and IL-18 detection

Following the centrifugation of ~ 2 mL peripheral blood samples collected from study subjects, serum was collected from the supernatant fraction. Serum concentrations of NLRP3, IL-1β, and IL-18 were then measured using appropriate commercial ELISA kits (Shanghai Jinma, China).

### Statistical analysis

Data were analyzed using SPSS 20.0. Continuous data were compared with Student’s t-tests or Mann-Whitney U tests. SNPs from both the case and control groups were assessed for Hardy-Weinberg equilibrium (HWE). Genotype and allele distribution frequencies for rs10754558 and rs10733113 were compared between case and control groups with Pearson chi-square tests, while the relationships between genotypes or alleles and clinical parameters were assessed through chi-square tests and logistic regression analyses. The relative risk associated with different alleles and genotypes was assessed based on calculated odds ratios (ORs) and 95% confidence intervals (95% CIs). P < 0.05 was the significance threshold.

## Results

### Baseline characteristics

Study participant characteristics at baseline are compiled in Table 2. No significant differences in age were observed when comparing cases and controls (P > 0.05). Of the included CC patients, 171(75.3%) and 56 (24.7%) were respectively classified as having squamous carcinoma and adenocarcinomas, respectively. Additionally, 143 (63.0%) and 84 (37.0%) CC patients had stage I/II and stage III/IV disease, while 185 (81.5%)and 42 patients (18.5%) respectively exhibited low and high levels of tumor differentiation. Of these patients, 31 (13.7%) exhibited lymph node metastases, while the remaining 196 (86.3%) were free of these metastases.

**Table 2:** Study subject characteristics

Characteristics	CC(n = 227)	CIN(n = 177)	Control (n = 419)	P1	P2	P3
**Age, years(mean±SD)**	51.93 ± 9.05	51.42 ± 7.31	50.88 ± 9.11	0.162	0.439	0.538
**Pathological types, n(%)**						
Squamous carcinoma	171(75.3)	-	-			
Adenocarcinoma	56(24.7)	-	-			
**TNM stage,n(%)**						
I/II	143(63.0)	-	-			
III/IV	84(37.0)	-	-			
**Differentiated degree,n(%)**						
Low	185(81.5)	-	-			
High	42(18.5)	-	-			
**Lymph node metastasis,n(%)**						
Yes	31(13.7)	-	-			
No	196(86.3)	-	-			

CC, cervical cancer; CIN, cervical intraepithelial neoplasia

P value (P1, comparison of CC with controls; P2, comparison of CIN with controls; P3, comparison of CC with CIN)

P value < 0.05 is considered statistically signifificant

### The relationship between NLRP3 genotype and allele frequencies and HPV infection status

**Fig. 3:**
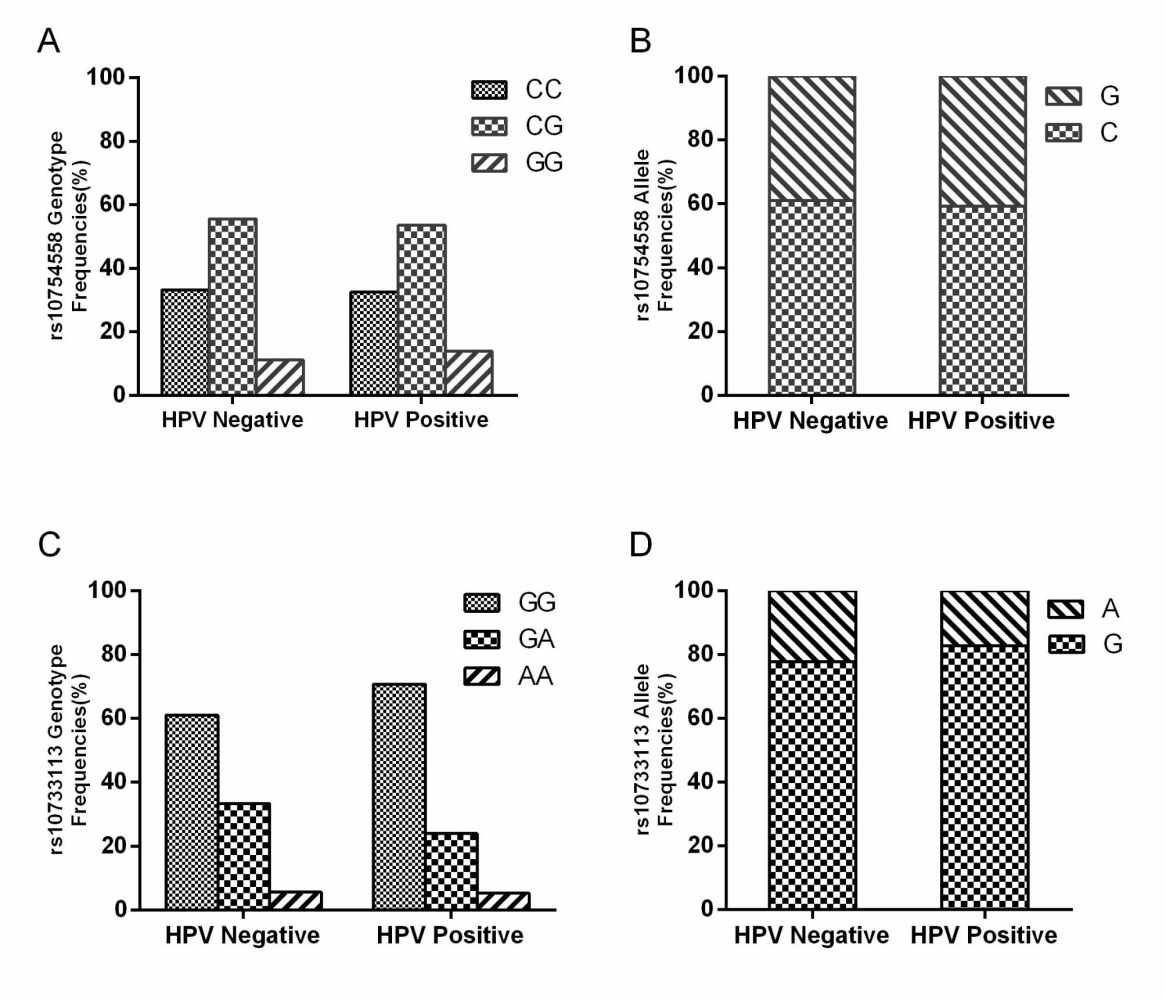
The rs10754558 genotype and allele frequencies in relation to HPV infection (**A**, **B**). The rs10733113 genotype and allele frequencies in relation to HPV infection (**C**, **D**)

Of the included cervical cancer cases, HPV infection was evident in 92.07% of cases(209/227), and no significant difference was observed among rs10754558 and rs10733113 genotype or allele frequencies with respect to HPV infection status (all P > 0.05) (Fig. 3).

### NLRP3 genotype and allele distribution frequency analyses

The HWE test revealed that the genotype frequencies for the rs10754558 and rs10733113 polymorphisms in both cases and controls conformed to the expected HWE (all P > 0.05). Then, genotype and allele distributions for these polymorphisms were assessed in cases and controls (Table 3). The rs10754558 GG genotype, CG + GG genotype, and G allele frequencies differed significantly between cases (CC and CIN) and healthy controls. Relative to patients with the CC genotype, those with the GG and CG + GG genotypes exhibited respective OR(95% CI) values of 1.815(1.129–2.916, P = 0.013) and 1.383(1.039–1.841, P = 0.026). Relative to the C allele, the G allele was associated with an OR(95% CI) of 1.284 (1.052–1.569, P = 0.014). In contrast, rs10733113 genotype and allele frequencies were not statistically significant when comparing the cases and controls (all P > 0.05).

**Table 3:** Genotype and allele distribution frequencies in cases and controls

SNP	Genotype	Cases(n = 404)	Controls(n = 419)	P value	OR (95%CI)
rs10754558	CC	130(32.18)	166(39.62)		1.000
	CG	220(54.45)	215(51.31)	0.077	1.307(0.971–1.758)
	GG	54(13.37)	38(9.07)	**0.013**	1.815(1.129–2.916)
	CG + GG	274(67.82)	253(60.38)	**0.026**	1.383(1.039–1.841)
	GG	54(13.37)	38(9.07)		1.000
	CG + CC	350(86.63)	381(90.93)	0.052	0.646(0.416–1.003)
	C Allele	480(59.41)	547(65.27)		1.000
	G Allele	328(40.59)	291(34.73)	**0.014**	1.284(1.052–1.569)
rs10733113	GG	279(69.06)	291(69.45)		1.000
	GA	104(25.74)	106(25.30)	0.886	1.023(0.746–1.404)
	AA	21(5.20)	22(5.25)	0.989	0.996(0.536-0.851)
	GA + AA	125(30.94)	128(30.55)	0.903	1.019(0.757–1.370)
	AA	21(5.20)	22(5.25)		1.000
	GA + GG	383(94.80)	397(94.75)	0.973	1.011(0.547–1.868)
	G Allele	662(81.93)	688(82.10)		1.000
	A Allele	146(18.07)	150(17.90)	0.929	1.012(0.787–1.301)

Cases, including cervical cancer and cervical intraepithelial neoplasia

### Age-stratified analyses of genotype distributions

Next, individuals in the case and control groups were stratified based on age into those ≤ 45 years old and those > 45 years old (Table 4). Among individuals > 45 years old, the rs10754558 GG genotype was associated with a significantly elevated risk of cervical lesions relative to the CC genotype(OR = 1.848, 95%CI: 1.023–3.339, P = 0.040), whereas the same was not true in individuals ≤ 45years old. No differences in rs10733113 genotype frequencies were observed when comparing cases and controls in either of these age groups (all P > 0.05).

**Table 4:** Analyses of NLRP3 polymorphism genotype distributions by age

Age genotype	Cases(n = 404)	Controls(n = 419)	P value	OR(95%CI)
rs10754558				
≤ 45 years old	n = 128	n = 135		
CC	47(36.72)	57(42.22)		1.000
CG	62(48.44)	64(47.41)	0.544	1.175(0.698–1.978)
GG	19(14.84)	14(10.37)	0.215	1.646(0.746–3.630)
CG + GG	81(63.28)	78(57.78)	0.362	1.259(0.767–2.068)
GG	19(14.84)	14(10.37)		1.000
CG + CC	109(85.16)	121(89.63)	0.274	0.664(0.318–1.387)
> 45 years old	n = 276	n = 284		
CC	86(31.16)	109(38.38)		1.000
CG	155(56.16)	151(53.17)	0.152	1.301(0.907–1.866)
GG	35(12.68)	24(8.45)	**0.040**	1.848(1.023–3.339)
CG + GG	190(68.84)	175(61.62)	0.073	1.376(0.970–1.952)
GG	35(12.68)	24(8.45)		1.000
CG + CC	241(87.32)	260(91.55)	0.103	0.636(0.367-1.100)
rs10733113				
≤ 45 years old	n = 128	n = 135		
GG	85(66.41)	82(60.74)		1.000
GA	38(29.69)	46(34.07)	0.397	0.797(0.471–1.348)
AA	5(3.91)	7(5.19)	0.537	0.689(0.210–2.258)
GA + AA	43(33.59)	53(39.26)	0.885	0.965(0.593–1.570)
AA	5((3.91)	7(5.19)		1.000
GG + GA	123(96.09)	128(94.81)	0.619	1.345(0.416–4.352)
> 45 years old	n = 276	n = 284		
GG	194(70.29)	209(73.59)		1.000
GA	66(23.91)	60(21.13)	0.406	1.185(0.794–1.769)
AA	16(5.80)	15(5.28)	0.709	1.149(0.553–2.387)
GA + AA	82(29.71)	75(26.41)	0.384	1.178(0.814–1.704)
AA	16(5.80)	15(5.28)		1.000
GG + GA	260(94.20)	269(94.72)	0.790	0.906(0.439–1.870)

### NLRP3 genotype and allele frequency distributions in patients with different types of cervical lesions

Patients in the case group were next separated into 227 CC patients and 177 CIN patients based on the grading of these cervical lesions, after which the relationships between NLRP3 polymorphism frequency distributions and CC or CIN incidence were assessed (Table 5). These analyses revealed that the frequencies of the rs10754558 GG genotype and G allele in the CC group were significantly elevated relative to the control group. Relative to the CC genotype, the GG genotype was associated with a significantly higher risk of developing CC (OR = 1.830, 95%CI: 1.058–3.165, P = 0.029), and the G allele was similarly associated with a higher risk than the C allele (OR = 1.281, 95%CI: 1.012–1.621, P = 0.039). In contrast, the rs10733113 genotype and allele frequencies were not significantly correlated with CC risk. Neither of these polymorphisms was significantly associated with CIN incidence.

**Table 5:** NLRP3 polymorphism genotype and allele distribution frequencies in the control, CC, and CIN groups

SNP	Genotype	Controls(n = 419)	CC(n = 227)	P1	OR (95%CI)	CIN(n = 177)	P2	OR (95%CI)
rs10754558	CC	166(39.62)	74(32.60)		1.000	56(31.64)		1.000
	CG	215(51.31)	122(53.74)	0.180	1.273(0.895–1.811)	98(55.37)	0.126	1.351(0.919–1.987)
	GG	38(9.07)	31(13.66)	**0.029**	1.830(1.058–3.165)	23(12.99)	0.054	1.794(0.985–3.269)
	CG + GG	253(60.38)	153(67.40)	0.078	1.357(0.966–1.905)	121(68.36)	0.066	1.418(0.977–2.057)
	GG	38(9.07)	31(13.66)		1.000	23(12.99)		1.000
	CC + CG	381(90.93)	196(86.34)	0.072	0.631(0.381–1.045)	154(87.00)	0.149	0.668(0.385–1.158)
	C Allele	547(65.27)	270(59.47)		1.000	210(59.32)		1.000
	G Allele	291(34.73)	184(40.53)	**0.039**	1.281(1.012–1.621)	144(40.68)	0.051	1.289(0.999–1.664)
rs10733113	GG	291(69.45)	159(70.04)		1.000	120(67.80)		1.000
	GA	106(25.30)	56(24.67)	0.861	0.976(0.663–1.410)	48(27.12)	0.648	1.098(0.735–1.641)
	AA	22(5.25)	12(5.29)	0.996	0.998(0.481–2.070)	9(5.08)	0.984	0.992(0.444–2.217)
	GA + AA	128(30.55)	68((29.96)	0.876	0.972(0.684–1.382)	57(32.20)	0.690	1.080(0.740–1.575)
	AA	22(5.25)	12(5.29)		1.000	9(5.08)		1.000
	GG + GA	397(94.75)	215(94.71)	0.985	0.993(0.482–2.045)	168(94.92)	0.934	1.034(0.467–2.293)
	G Allele	688(82.10)	374(82.38)		1.000	288(81.36)		1.000
	A Allele	150(17.90)	80(17.62)	0.901	0.981(0.727–1.323)	66(18.64)	0.760	1.051(0.763–1.448)

P value (P1, comparison of CC with controls; P2, comparison of CIN with controls)

### The relationship between NLRP3 genotype frequencies and cervical cancer pathological characteristics

Next, associations between NLRP3 polymorphism genotype frequencies and tumor pathological characteristics were assessed in the CC patient cohort (Table 6). No significant relationships were detected between the rs10754558 or rs10733113 genotype frequencies and CC patient pathological characteristics (all P > 0.05).

**Table 6:** Associations between NLRP3 polymorphisms and CC patient pathological characteristics

SNP	Pathological parameters	Genotype				P value	OR (95%CI)
rs10754558		CC n(%)	CG n(%)	GG n(%)			
	**Pathological types**						
	Squamous carcinoma(n = 171)	55(32.16)	93(54.39)	23(13.45)	CCvs.CG	0.764	0.903(0.463–1.760)
	Adenocarcinoma(n = 56)	19(33.93)	29(51.78)	8(14.29)	CCvs.GG	0.989	1.007(0.386–2.626)
	**TNM stage**						
	I/II(n = 143)	50(34.96)	76(53.15)	17(11.89)	CCvs.CG	0.455	1.261(0.686–2.319)
	III/IV(n = 84)	24(28.57)	46(54.76)	14(16.67)	CCvs.GG	0.216	1.716(0.727–4.049)
	**Differentiated degree**						
	Low(n = 185)	63(34.05)	100(54.05)	22(11.89)	CCvs.CG	0.566	1.260(0.572–2.775)
	High(n = 42)	11(26.19)	22(52.38)	9(21.43)	CCvs.GG	0.092	2.343(0.857–6.406)
	**Lymph node metastasis**						
	Yes(n = 31)	10(32.26)	17(54.84)	4(12.90)	CCvs.CG	0.934	0.965(0.416–2.237)
	No(n = 196)	64(32.65)	105(53.57)	27(13.78)	CCvs.GG	0.933	1.055(0.304–3.658)
rs10733113		GG n(%)	GA n(%)	AA n(%)			
	**Pathological types**						
	Squamous carcinoma(n = 171)	119(69.59)	43(25.15)	9(5.26)	GGvs.GA	0.772	0.899(0.439–1.841)
	Adenocarcinoma(n = 56)	40(71.43)	13(23.21)	3(5.36)	GGvs.AA	0.990	0.992(0.256–3.844)
	**TNM stage**						
	I/II(n = 143)	98(68.53)	37(25.87)	8(5.60)	GGvs.GA	0.555	0.825(0.435–1.563)
	III/IV(n = 84)	61(72.62)	19(22.62)	4(4.76)	GGvs.AA	0.729	0.803(0.232–2.782)
	**Differentiated degree**						
	Low(n = 185)	129(69.73)	47(25.41)	9(4.86)	GGvs.GA	0.640	0.823(0.364–1.863)
	High(n = 42)	30(71.43)	9(21.43)	3(7.14)	GGvs.AA	0.604	1.433(0.366–5.616)
	**Lymph node metastasis**						
	Yes(n = 31)	21(67.74)	9(29.03)	1(3.23)	GGvs.GA	0.595	0.795(0.340–1.856)
	No(n = 196)	138(70.41)	47(23.98)	11(5.61)	GGvs.AA	0.627	1.674(0.205–13.642)

### Measurement of serum NLRP3, IL-1β, and IL-18 concentrations

**Fig. 4:**
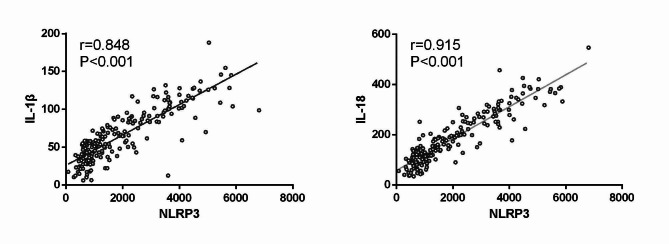
Correlations between serum NLRP3 levels and levels of IL-1β and IL-18 in CC patients

Patients in the CC group exhibited significantly higher serum NLRP3, IL-1β, and IL-18 levels relative to controls (all P < 0.05) (Table 7). Correlation analyses indicated that NLRP3 levels were positively correlated with levels of both IL-1β (r = 0.848, P < 0.001) and IL-18 (r = 0.915, P < 0.001) in the CC group (Fig. 4).

**Table 7:** Serum NLRP3, IL-1β, and IL-18 concentrations in the CC and control groups

Index	CC(n = 227)	Controls(n = 419)	P value
NLRP3(pg/ml)	1459.4(820.6,3052.5)	1020.6(681.7,2087.5)	0.000
IL-1β(pg/ml)	58.4(39.1,90.7)	50.12(31.5,72.9)	0.002
IL-18(ng/L)	154.8(110.5,252.3)	134.0(90.3,215.4)	0.006

### The relationship between NLRP3 genotypes and serum NLRP3, IL-1β, and IL-18 concentrations

As shown in Table 8, a significant correlation was observed between the rs10754558 GG genotype and serum IL-1β and IL-18 levels in CC patients (both P < 0.05), with levels of both of these cytokines being elevated as compared to patients with the CC genotype. In contrast, rs10754558 genotype was unrelated to NLRP3 levels. Patient with rs10733113 genotype was also unrelated to serum NLRP3,IL-1β, or IL-18 levels.

**Table 8:** The relationship between NLRP3 genotypes and serum concentrations of NLRP3, IL-1β, and IL-18 levels in CC

SNP	Index		Genotype			P value
rs10754558		CC(n = 74)	CG(n = 122)	GG(n = 31)		
	NLRP3(pg/ml)	1378.9(778.9,2851.1)	1417.8(828.9,2641.4)	2281.7(917.8,3738.6)	CCvs.CG	0.778
					CCvs.GG	0.158
	IL-1β (pg/ml)	50.3(31.2,83.3)	58.8(43.0,88.9)	85.0(46.0,104.0)	CCvs.CG	0.074
					CCvs.GG	**0.014**
	IL-18(ng/L)	139.9(86.9,257.1)	149.9(115.0,235.9)	239.8(132.3,308.5)	CCvs.CG	0.461
					CCvs.GG	**0.032**
rs10733113		GG (n = 159)	GA (n = 56)	AA (n = 12)		
	NLRP3(pg/ml)	1462.2(834.4,3092.8)	1290.0(734.4,2434.4)	2506.7(842.8,3923.3)	GGvs.GA	0.513
					GGvs.AA	0.461
	IL-1β (pg/ml)	55.4(35.4, 90.6)	61.6(50.1,86.6)	71.9(30.1,106.9)	GGvs.GA	0.191
					GGvs.AA	0.692
	IL-18(ng/L)	151.5(103.8,251.9)	161.7(121.9,250.3)	240.4(97.4,328.8)	GGvs.GA	0.451
					GGvs.AA	0.413

## Discussion

Cervical cancer remains a major threat to the health of women, and its onset is driven by complex interactions between environmental and genetic factors. SNPs are the most common and best-studied type of genetic mutation, and specific SNPs have thus been explored as diagnostic biomarkers, prognostic biomarkers, and therapeutic targets in CC and many other diseases [19]. Inflammation is also a key driver of CC, with Deivendran et al. [20] having determined that inflammatory activity occurring following viral infection serves as a particularly important factor that can stimulate oncogenic growth and development. Infiltrating immune cells can secrete a range of growth factors and chemokines in cervical neoplasms that contribute to enhanced malignancy. This study was thus designed to focus on the impact of polymorphisms in the NLRP3 gene on HPV infection and CC risk, revealing the rs10754558 polymorphism to ultimately be associated with higher risk of CC in southern Chinese population.

The NLRP3 inflammasome can be activated in response to a range of damage- and pathogen-associated molecular patterns, resulting in the maturation and release of important pro-inflammatory cytokines including IL-1β and IL-18 that activate inflammatory signaling cascades in addition to the induction of pyroptotic cell death [21]. NLPR3 signaling activity has been closely linked to a range of disease states such as autoimmunity, cardiovascular disease, chronic obstructive pulmonary disease, and various malignancies [22–25]. The NLRP3 inflammasome can also promote the onset of diverse cancer types, impacting important processes including proliferative, angiogenic, metastatic, and immunosuppressive activity. For example, in one report NLRP3 inflammasome activation was shown to promote autocrine IL-1β secretion in breast cancer, in turn inducing the epithelial-mesenchymal transition and metastatic progression [26]. In a separate report, macrophages were found to promote the metastatic progression of colorectal cancer through mechanisms dependent on the activity of the NLRP3 inflammasome [27]. Boone et al. determined that in a mouse pancreatic cancer model system, increased platelet NLRP3 inflammasome activity is linked to platelet aggregation and tumor growth [28]. There is also evidence for the ability of NLRP3 signaling to contribute to the establishment of an immunosuppressive microenvironment in pancreatic cancer by promoting T cells to undergo tolerogenic differentiation and through the IL-10-mediated suppression of adaptive immunity [29]. However, some studies have found that NLRP3 can suppress the growth of certain tumor types. For example, Dupaul et al. determined that colorectal cancer metastasis to the liver could be inhibited by the NLRP3 inflammasome through the induction of NK cell-mediated tumor killing [30]. Separately, the antitumor drug Alpinumisoflavone was found to suppress the growth and metastasis of hepatocellular carcinoma cells through the induction of pyroptosis in an NLRP3 inflammasome-dependent fashion [31]. The NLRP3 inflammasome may thus play beneficial and deleterious roles in specific cancers in a context-dependent manner that may be influenced by factors including downstream effector molecule expression, tissue or cell type, the stage of tumor progression, and the impact of particular genetic mutations on the expression or function of NLRP3.

Recent studies have sought to clarify the mechanisms through which the NLRP3 inflammasome may influence the development and progression of CC. Pathogen-derived ligands can trigger oncogenesis through interactions with their cognate receptors, as with the binding of LPS to Toll-like receptor 4. Researchers have previously shown that LPS-stimulated human SiHa and Caski CC cells, which are infected with HPV-16, exhibit the upregulation of key inflammasome-related proteins including NLRP3, pro-IL-1β, IL-1β, and caspase-1. When inflammatory activity persists for extended periods, this can drive normal cervical cells to undergo malignant transformation while establishing a microenvironment conducive to tumor growth [32]. Here, serum NLRP3, IL-1β, and IL-18 levels were found to be significantly elevated in CC patients relative to controls, with NLRP3 levels being significantly positively correlated with the levels of IL-1β and IL-18, supporting a potential relationship between the NLRP3 inflammasome and CC. Polymorphisms in the NLRP3 gene may also play a key role in increasing patient susceptibility to CC development.

SNPs are the most common type of genetic variant, and advances in SNP probe technologies and genomics strategies have led to the identification of links between SNPs and specific diseases. Over 60 human NLRP3 gene SNPs have been identified to date, with these mutations being most common within the nucleotide oligomerization domain (NACTH) region of the protein. These variations can result in changes in the structure of the NLRP3 protein such that it is constitutively active, resulting in persistent NF-κB and caspase-1 activation, in turn contributing to excessive inflammatory cytokine production and overly exuberant immune cell activity [33]. NLRP3 SNPs have also been linked to particular inflammatory disease states. For example, the rs10157379 CT and rs10754558 GG genotypes were recently linked to COVID-19-related inflammation [14]. Additionally, Keskin et al. identified NLRP3 polymorphisms as important biomarkers associated with bone resorption in the context of chronic otitis media [34], while Slezakova et al. detected a link between the NLRP3 rs4612666 polymorphism and recurrent aphthous stomatitis (RAS) incidence in a Czech cohort [35]. However, there have only been a limited number of studies examining the association between mutations in the NLRP3 gene and cancer incidence. The rs35829419 polymorphism has been found to be related to poorer invasive CRC patient survival [36], and it has thus been suggested to offer value as a prognostic biomarker in CRC [37]. In Chinese individuals, the rs35829419 polymorphism has been linked with elevated head and neck cancer risk [38]. The specific associations between the rs10754558 and rs10733113 NLRP3 polymorphisms and CC, in contrast, have yet to be characterized.

Here, the rs10754558 GG genotype, CG + GG genotype, and G allele were all found to be associated with a higher risk of cervical lesion development, with the GG genotype in particular being significantly associated with higher odds of cervical lesion development among patients over the age of 45. Whereas Liu et al. revealed the fact that peak cervical lesion incidence in mainland China occurs between the ages of 30 and 50 [39]. Further analyses revealed significant associations between CC risk and both the GG genotype and G allele, whereas no correlation was detected between the rs10754558 polymorphism and patient clinicopathological parameters or HPV infection status. This differs from prior work, as Pontillo et al. found a significant association between rs10754558 and both high-risk HPV infection and HPV persistence [40]. The rs10754558 polymorphism has been studied in great detail in prior work and found to be closely related to the incidence of a range of complex diseases. For example, rs10754558 polymorphisms have been linked to ischemic stroke risk and preeclampsia incidence among Chinese individuals [41, 42]. Ehtesham et al. also found rs10754558 to be associated with the risk of developing systemic lupus erythematosus and the severity of disease in patients with this form of autoimmunity [43]. Studies examining the association between this rs10754558 polymorphism and tumors, however, have been less common. In one report, patients carrying the rs10754558 polymorphism were found to be at a higher risk of developing gastric cancer following *Helicobacter pylori* infection [12], and in another report, it was linked to higher bladder cancer risk, particularly among smokers and individuals that drink alcohol. Moreover, in bladder cancer patients rs10754558 has been linked to both tumor size and lymph node metastasis [16]. In contrast, there have been fewer studies examining the association between the NLRP3 rs10733113 polymorphism and disease. However, rs10733113 has been linked to elevated Crohn’s disease risk, and the rs10733113 G allele is reportedly associated with a reduction in the need for surgery and in maximal disease activity in patients with Crohn’s disease [17]. The rs10733113 G allele is also reportedly associated with an early-onset skin disease in psoriatic arthritis patients [18]. No studies, however, have examined the association between this rs10733113 polymorphism and tumors to date, and this is the first report to have examined its relationship with CC. These analyses did not detect any significant correlative relationship between rs10733113 genotype or allele frequencies and CC susceptibility in the analyzed participant cohort.

Both IL-1β and IL-18 are major inflammatory cytokines capable of inducing the production of a range of chemotactic factors and adhesion molecules that can potentiate inflammatory activity and promote inflammatory cell infiltration. In tumor cells, IL-1β can promote NF-kB pathway activation, tumor cell proliferation, and epithelial-mesenchymal transition activity in a manner that contributes to tumor progression [44]. IL-18 is related to cancer onset and patient clinical outcomes through its ability to promote the activation of immune cells such as NK and T cells, inducing IFN-γ secretion and driving enhanced cytotoxic activity [45]. Here, serum NLRP3, IL-1β, and IL-18 levels were compared among different genotypes of CC patients, revealing significant increases in serum IL-1β and IL-18 levels in patients with the rs10754558 GG genotype relative to those with the CC genotype. Accordingly, the rs10754558 G allele may alter the function of NLRP3, thereby influencing IL-1β and IL-18 secretion in a manner that enhances inflammatory cascade activity and drives pathological changes conducive to cervical oncogenesis.

This study is subject to some limitations. For one, only two NLRP3 SNPs were analyzed in this patient population, and studies of other polymorphisms are warranted. Second, the mechanisms whereby rs10754558 polymorphisms contribute to a higher risk of CC remain to be clarified. Third, this was a case-control study that is thus subject to inevitable selection bias. Lastly, this was a single-center study, and additional large-scale multicenter studies will be necessary to fully elucidate the link between NLRP3 polymorphisms on CC susceptibility.

In summary, these results are the first to show a significant association between the NLRP3 rs10754558 polymorphism and an elevated risk of CC in southern Chinese population. Future studies should therefore seek to clarify the mechanistic basis for this risk relationship and to further examine the precise association between NLRP3 gene variants and this deadly gynecological malignancy.

### Data Availability

The raw data supporting the results and conclusions of this article will be made available by the authors. All data generated or analyzed presented in the study are included in the article.
